# *Serping1/*C1 Inhibitor Affects Cortical Development in a Cell Autonomous and Non-cell Autonomous Manner

**DOI:** 10.3389/fncel.2017.00169

**Published:** 2017-06-16

**Authors:** Anna Gorelik, Tamar Sapir, Trent M. Woodruff, Orly Reiner

**Affiliations:** ^1^Department of Molecular Genetics, Weizmann Institute of ScienceRehovot, Israel; ^2^School of Biomedical Sciences, The University of QueenslandSt Lucia, QLD, Australia

**Keywords:** Serping1, C1 inhibitor, innate immune complement pathway, neuronal migration, neuronal stem cell proliferation

## Abstract

Current knowledge regarding regulation of radial neuronal migration is mainly focused on intracellular molecules. Our unbiased screen aimed at identification of non-cell autonomous mechanisms involved in this process detected differential expression of *Serping1* or C1 inhibitor, which is known to inhibit the initiation of the complement cascade. The complement cascade is composed of three pathways; the classical, lectin, and the alternative pathway; the first two are inhibited by C1 inhibitor, and all three converge at the level of C3. Knockdown or knockout of *Serping1* affected neuronal stem cell proliferation and impaired neuronal migration in mice. Knockdown of *Serping1* by *in utero* electroporation resulted in a migration delay of the electroporated cells as well as their neighboring cells demonstrating a non-cell autonomous effect. Cellular polarity was also affected. Most importantly, expression of protein components mimicking cleaved C3 rescued the knockdown of *Serping1*, indicating complement pathway functionality. Furthermore, we propose that this activity is mediated mainly via the complement peptide C5a receptors. Whereas addition of a selective C3a receptor agonist was minimally effective, the addition of a dual C3aR/C5a receptor agonist significantly rescued *Serping1* knockdown-mediated neuronal migration defects. Our findings suggest that modulating *Serping1* levels in the developing brain may affect the complement pathway in a complex way. Collectively, our findings demonstrate an unorthodox activity for the complement pathway during brain development.

## Introduction

Deciphering what is the function of molecules expressed in the developing brain is a daunting task. Therefore, several years ago we embarked on an unbiased functional screen aimed at detecting molecules, which may affect neuronal migration in a non-cell autonomous way (Greenman et al., 2015). One of the differentially expressed genes detected in this screen was *Serping1* (Serpin peptidase inhibitor, clade G, member 1) encoding for the C1 inhibitor protein. C1 inhibitor is a member of the serpin family of protease inhibitors (reviewed by Davis et al., 2008). Similar to other serpin family protease inhibitors, the mechanism of inhibition requires a physical contact between the inhibitor and a specific protease, followed by a conformational change and formation of a covalent bond between the inhibitor and the serine residue which is part of the protease active site. C1 inhibitor has a key role in the complement pathway where it inhibits initiation proteases in either the classical pathway (C1r and C1s) or the lectin pathway (MASP1 and MASP2) (Presanis et al., 2003; Parej et al., 2013). C1 inhibitor has additional important substrates that include contact system proteases (factor XII, plasma kallikrein), an intrinsic coagulation protease (factor XI) and the fibrinolytic proteases (plasmin, tissue plasminogen activator). However, based on our recent studies demonstrating the expression and function of the complement system in brain development (Coulthard et al., 2017; Gorelik et al., 2017), the current study on the role of *Serping1* in the developing brain has been focused on its relationship within the complement pathway. In addition to protease inhibition, C1 inhibitor can physically bind and functionally affect the interaction between complement factor C3b and complement factor B and thus to interfere also with the alternative pathway (Jiang et al., 2001). Additional functional interactions include different extracellular matrix components, endothelial cells and leukocytes, gram negative endotoxin, and several infectious agents (reviewed by Davis et al., 2008).

C1 inhibitor has been associated with several diseases. Addition of C1 inhibitor has been shown to be neuroprotective in case of ischemic injury (De Simoni et al., 2004; Storini et al., 2005; Gesuete et al., 2009; Heydenreich et al., 2012). However, it is likely that the neuroprotection is not mediated solely via the activity of C1 inhibitor on the complement pathway. Expression of multiple components of the complement pathway, including C1 inhibitor has also been demonstrated in Alzheimer's disease, which may reflect ongoing inflammation in the brains of the patients (Walker et al., 1995; Veerhuis et al., 1998; Yasojima et al., 1999). It has been suggested that reduced levels of C1 inhibitor may be a biomarker for Alzheimer's disease (Akuffo et al., 2008; Cutler et al., 2008; Chiam et al., 2015; Muenchhoff et al., 2015; Morgan et al., 2017).

Deficiency of C1 inhibitor is a rare autosomal dominant disease known as Hereditary angioedema (HAE) with an estimated prevalence of 1:50,000, where about 25% of the patients exhibit *de novo* mutations (Bowen et al., 2010). Patients with HAE may experience recurrent edema of the skin and submucosal tissue associated with pain syndromes, nausea, vomiting, diarrhea, and life-threatening airway swellings. Risk of dying from airway obstruction if left untreated is significant. Additional symptoms may present as well and the manifestations and severity of HAE are highly variable. In this disease, the low levels of active C1 inhibitor in the plasma leads to unregulated activation of the complement and contact cascades and the development of angioedema with its associated complications. Complement system activation results in decreased levels of C4 and C2, while contact system activation results in cleavage of high molecular weight kininogen. Studies conducted in a mouse model for this disease revealed that both homozygous and heterozygous mice exhibit increased vascular permeability in comparison with wild-type littermates (Han, 2002). They have further shown that this phenotype is mediated through the bradykinin type 2 receptor.

In contrast to its roles in innate immunity, very little is known about the expression and functional activity of *Serping1* in the developing brain. A study examining single cell RNA expression in the E14 developing mouse brain revealed that *Serping1* is expressed in subventricular zone (SVZ) basal progenitors (Kawaguchi et al., 2008). In this study, we therefore set out to investigate the role of this interesting molecule in the developing cortex and how its function there relates to the complement pathway.

## Results

### *Serping1* is expressed in the developing brain

*Serping1* was detected in developing mouse brains (E14.5-E17.5) in an unbiased screen aimed at identifying molecules which may affect neuronal migration in a non-cell autonomous way (Greenman et al., 2015). The screen was designed to highlight changes between genes expressed in stalled cells that acquire either a bipolar (following *Dclk* shRNA treatment) or a multipolar appearance (following *Dclk* shRNA treatment). The results of the screen showed that the levels of *Serping1* were 2.10-fold higher in *Dclk* shRNA vs. *Dcx* shRNA. The differences at the mRNA level were verified by realtime qPCR (59.6 ± 1.5% in *Dcx* shRNA compared to *Dclk* shRNA, *n* = 6, *Student's t-test, p* = 0.0047, Supplementary Figure 1A) and were also recapitulated at the protein level, with an elevation of 159.6 ± 8.82% in SERPING1 protein in *Dclk* shRNA-treated brains vs. *Dcx* shRNA-treated brains (*n* = 5, *Student's t-test, p* = 0.041, Supplementary Figure 1A). Following these results we next examined *Serping1* mRNA expression in the developing cortex using real-time qPCR (Supplementary Figure 1B). When the expression was normalized to that observed on E13.5, similar levels were noted on E14.5, followed by an observed decreased expression on E16.5 and E18.5. RNA *in situ* hybridization data from E14.5 brain section taken from (http://www.genepaint.org) demonstrated that *Serping1* mRNA is expressed in the developing cortex, where the highest expression levels are seen in the SVZ as previously reported (Kawaguchi et al., 2008). However, in addition *Serping1* mRNA was expressed in the ventricular zone, and lower levels of expression could also be observed in the cortical plate (Supplementary Figure 1C). The timing and pattern of *Serping1* expression suggested that this gene may participate in neuronal stem cell proliferation.

### *Serping1* affects neuronal stem cell proliferation

**Figure 1:**
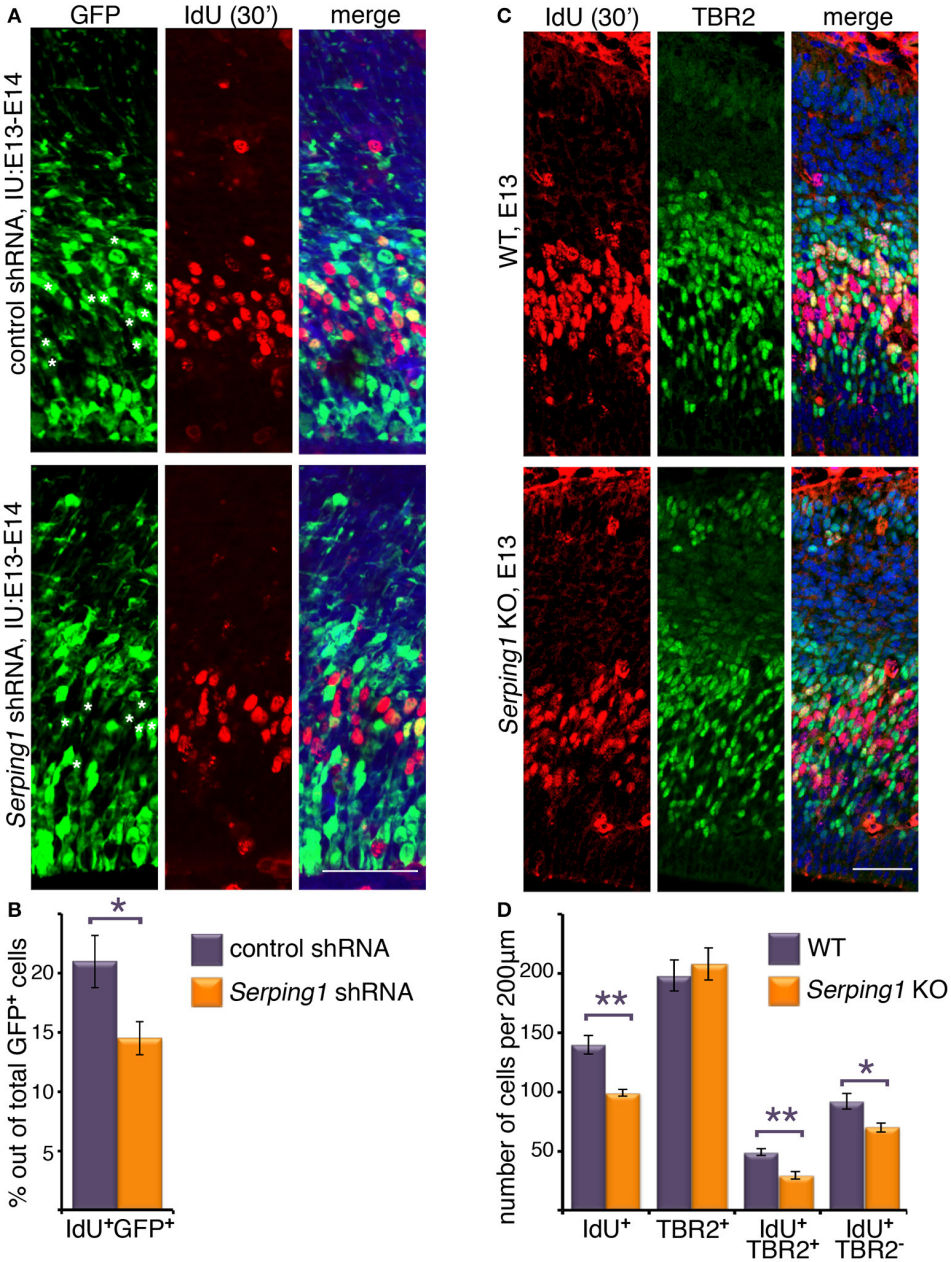
*Serping1* affects neuronal stem cell proliferation. **(A,B)** Embryonic brains were *in utero* electroporated with control shRNA or *Serping1* shRNA at E13 and at E14 were treated with IdU for 30 min. The brains were cryosectioned and immunostained with anti-IdU antibodies. GFP labeled the electroporated cells. IMARIS software was used to count the total GFP-positive cells and the double GFP- and IdU-positive cells within slices of the same size (230 μm in length). Double-positive cells are marked with white asterisks in the GFP panel. The relative proportion of double-positive cells to the total number of GFP-positive cells was calculated (**B**, *Student t*-test, *n* = 5, ^*^p < 0.05). **(C,D)** Brains of E13 *Serping1* KO and littermate WT were treated with IdU for 30 min. The brains were cryosectioned and immunostained with anti-IdU and anti-TBR2 antibodies. The number of IdU-positive, TBR2-positive, double positive, IdU-positive TBR2-negative cells was counted **(D)** in identical areas of the cortices (200 μm in length). *Welch's t*-test, *n* = 6, ^*^p < 0.05, ^**^p < 0.01. The scale bars are 50 μm.

To investigate the role of *Serping1* in regulation of neuronal stem cell proliferation during mouse embryonic brain development two models were used. In the first, we knocked-down gene expression using *in utero* electroporation of an shRNA expressing plasmid, and in the second we generated knockout embryos using CRISPR/Cas9 gene editing technology (Ran et al., 2013; Wang et al., 2013) (Supplementary Figures 2A,B). *Serping1* shRNA effectively reduced the levels of *Serping1* mRNA by 37.5 ± 8.9% in comparison to control (qPCR, *n* = 9, *Student's t-test p* = 0.00012), as well as SERPING1 protein in the developing brain (52.7 ± 5.6% compared to control, *n* = 4, *p* = 0.0064). Neuronal stem cell proliferation was tested by application of a short IdU pulse, which is incorporated during S phase following by immunostaining of embryonic brain sections using the respective antibodies. Embryos were *in utero* electroporated at E13 and analyzed at E14. The analysis was targeted at the IdU/GFP positive cells, which were likely to receive the respective shRNA plasmids. There was a statistically significant difference between the IdU/GFP double positive cells, where the introduction of *Serping1* shRNA reduced the number of cells in S-phase (*Student's t-test* 21 ± 2.2% vs. 14.5 ± 1.4, *n* = 5, *p* = 0.037, Figures 1A,B). In addition, embryos in which *Serping1* was knocked out were generated. Brain sections were immunostained for IdU and also for TBR2, a basal progenitors marker, using the respective antibodies (Figure 1C). Similar to the trend observed in case of *Serping1* knockdown, a significant reduction in the number of IdU positive cells was noted (*Student's t-test, Welch-corrected* 140 ± 8 vs. 99 ± 2.7, *n* = 6, *p* = 0.0028, control and *Serping1* KO, respectively, Figures 1C,D). Whereas, the total number of TBR2 positive cells did not differ between the wild-type and the knockout, there was a clear difference in the number of IdU/TBR2 positive cells, where less cells were double labeled in the KO (*Student's t-test*, 49 ± 3.13 vs. 29 ± 2.9, *n* = 5,6, respectively, *p* = 0.0012, Figures 1C,D), thus suggesting that in the *Serping1* KO there is a reduction of intermediate progenitors at E14. In addition, the number of IdU positive/TBR2 negative cells was also decreased in the *Serping1* KO (*Student's t-test*, 92.2 ± 6.8, *n* = 5 vs. 69.83 ± 3.85, *n* = 6, *p* = 0.0154, Figures 1C,D). To check whether the observed differences resulted from alterations in S phase duration, double labeling using two thymidine analogs was performed. Embryos were *in utero* electroporated at E13 with either control shRNA or *Serping1* shRNA. On E14 the cells were labeled with EdU for the duration of 2.5 h and then with IdU for 0.5 h (Supplementary Figure 3). No significant differences in the proportion of EdU/GFP double positive cells (14.1 ± 0.5% in *Serping1* shRNA vs. 16.5 ± 1.6% in control, *n* = 7, Sidak's multiple comparison test) or IdU/EdU/GFP triple positive cells (7.8 ± 0.8 vs. 10.6 ± 0.6%, *n* = 7) thus suggesting that the cell cycle length was not affected. The proportion of IdU/GFP double positive cells was reduced in *Serping1* shRNA consistent with previous experiments (17.4 ± 1.2% vs. 23.5 ± 0.9, *n* = 7). In addition significantly more GFP only positive cells were noted in case of *Serping1* shRNA treatment, suggesting that these cells were not actively in S-phase during the tested period (76.3 ± 1.1 vs. 70.6 ± 1.5%, *n* = 7). Collectively, our results suggest that knockdown or knockout of *Serping1* results in a reduction in the number of cycling cells of both ventricular zone (radial) and intermediate (basal) progenitors during cortical development.

### *Serping1* affects radial migration in a cell autonomous and non-cell autonomous way

**Figure 2:**
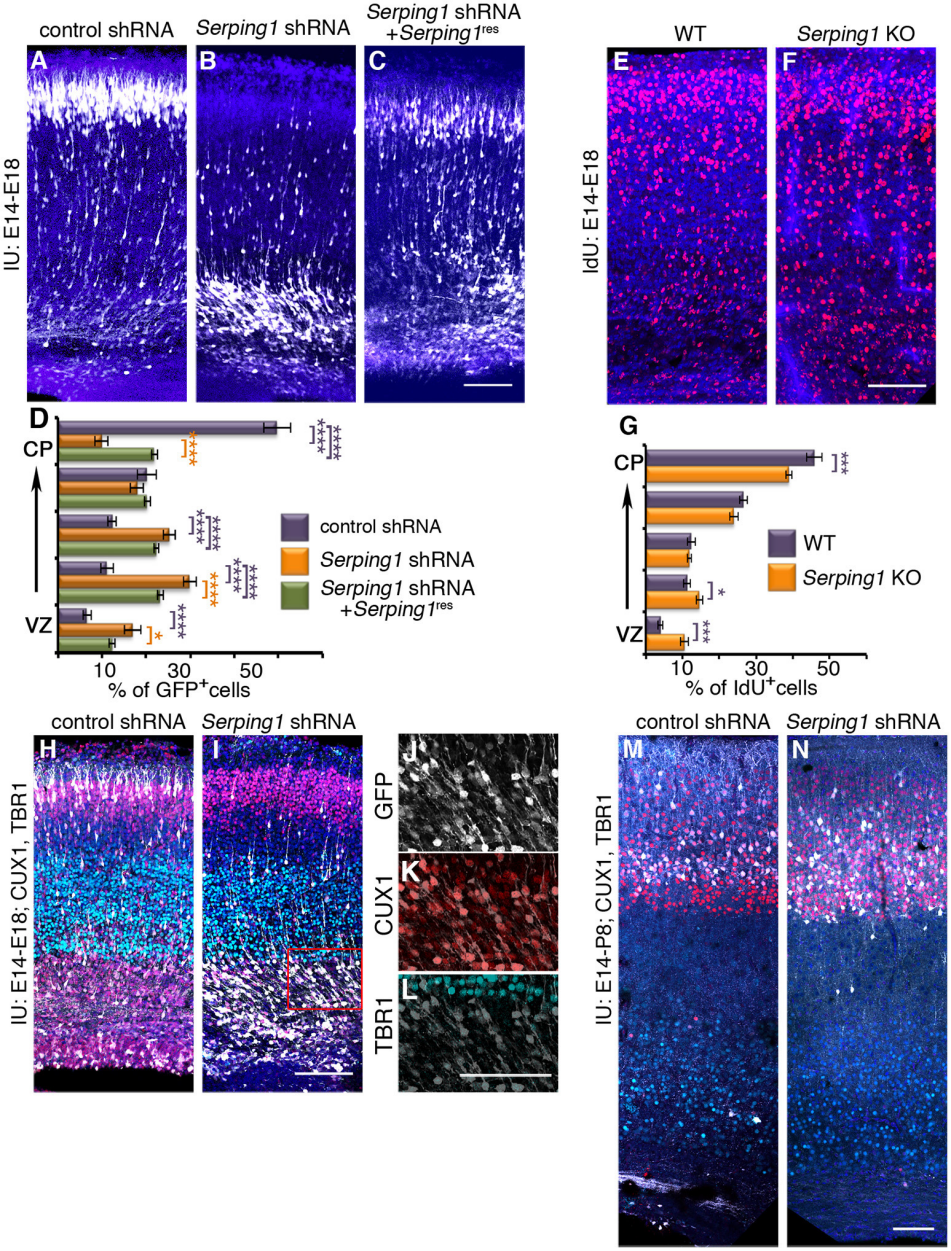
*Serping1* affects neuronal migration. **(A–D)** *Serping1* knockdown affects neuronal migration. Brains were electroporated *in utero* (E14-E18) with control shRNA **(A)**, *Serping1* shRNA alone **(B)** or in combination with *Serping1* resistant to the shRNA **(C)**. **(D)** The position of GFP+ neurons across the width of the cortex was analyzed and is shown in 5 bins (from the VZ to the CP). The statistical significance of comparison to control is shown in violet. The statistical significance of comparison to *Serping1* shRNA is shown in orange. Two-way ANOVA, *n* = 11, ^*^p < 0.05, ^****^p < 0.0001. **(E–G)** *Serping1* KO affects neuronal migration. *Serping1* KO embryos **(F)** and littermate controls **(E)** were labeled with IdU on E13 and the position of IdU-positive neurons was analyzed on E18 and is shown **(G)** in 5 bins (from the VZ to the CP). Two-way ANOVA, *n* = 12, ^*^p < 0.05, ^***^p < 0.001. **(H–L)** The identity of electroporated cells. Slices of control shRNA and *Serping1* shRNA were immunostained with anti-CUX1 (red) and anti-TBR1 (light green) antibodies. *Serping1* shRNA arrested cells are shown in higher magnification **(J–L)**. **(M–N)** Postnatal positioning and the identity of control and *Serping1* knockdown cells. Brains electroporated *in utero* on E14 with control shRNA **(M)** or *Serping1* shRNA **(N)** were immunostained at postnatal day 8 (P8) with anti-CUX1 (red) or anti-TBR1 (light-green) antibodies. The scale bars are 100 μm.

The possible role of *Serping1* in regulation of radial migration was investigated by knockdown of the gene using *in utero* electroporation of *Serping1* shRNA and by studying knockout embryos generated by CRISPR/Cas9 gene editing. *Serping1* shRNA significantly impaired radial neuronal migration (compare control in Figure 2A to Figure 2B), and this phenotype was partially rescued following the addition of *Serping1* shRNA-resistant form (*Serping1*^res^) (Figure 2C). The position of the GFP positive cells was quantified in five bins across the width of the cortex. A two-way ANOVA demonstrated that the number of cells in the different bins differed between the control and *Serping1* shRNA in four out of five bins (Figure 2D). Addition of *Serping1*^res^ restored the level of SERPING1 protein to control levels (data not shown) and significantly improved the position of the cells in three out of four bins (Figure 2D). Neuronal migration impairment was also detected in *Serping1* knockout embryos in comparison with wild type litter-mates (Figures 2E,F quantified in Figure 2G). Neurons were birth-dated with a uridine analog at E14.5 and their relative position in the cortex was scored at E18. The distribution of neurons along the width of the cortex significantly differed in three out of five bins (Figure 2G). Next, the identity of the stalled cells at E18 was examined (Figures 2H–L). Although most of the *Serping1* shRNA treated cells have not reached the cortical plate (compare the GFP+ cells in Figure 2I vs. Figure 2H), the majority express the superficial layer marker CUX1 and not the deep layer marker TBR1 (Figures 2J–L). In the postnatal brain (at P8), *Serping1* shRNA treated cells did reach the cortical plate, nevertheless, even in low magnifications, it is possible to observe that their morphology differs from that observed in the control (Figures 2M,N).

**Figure 3:**
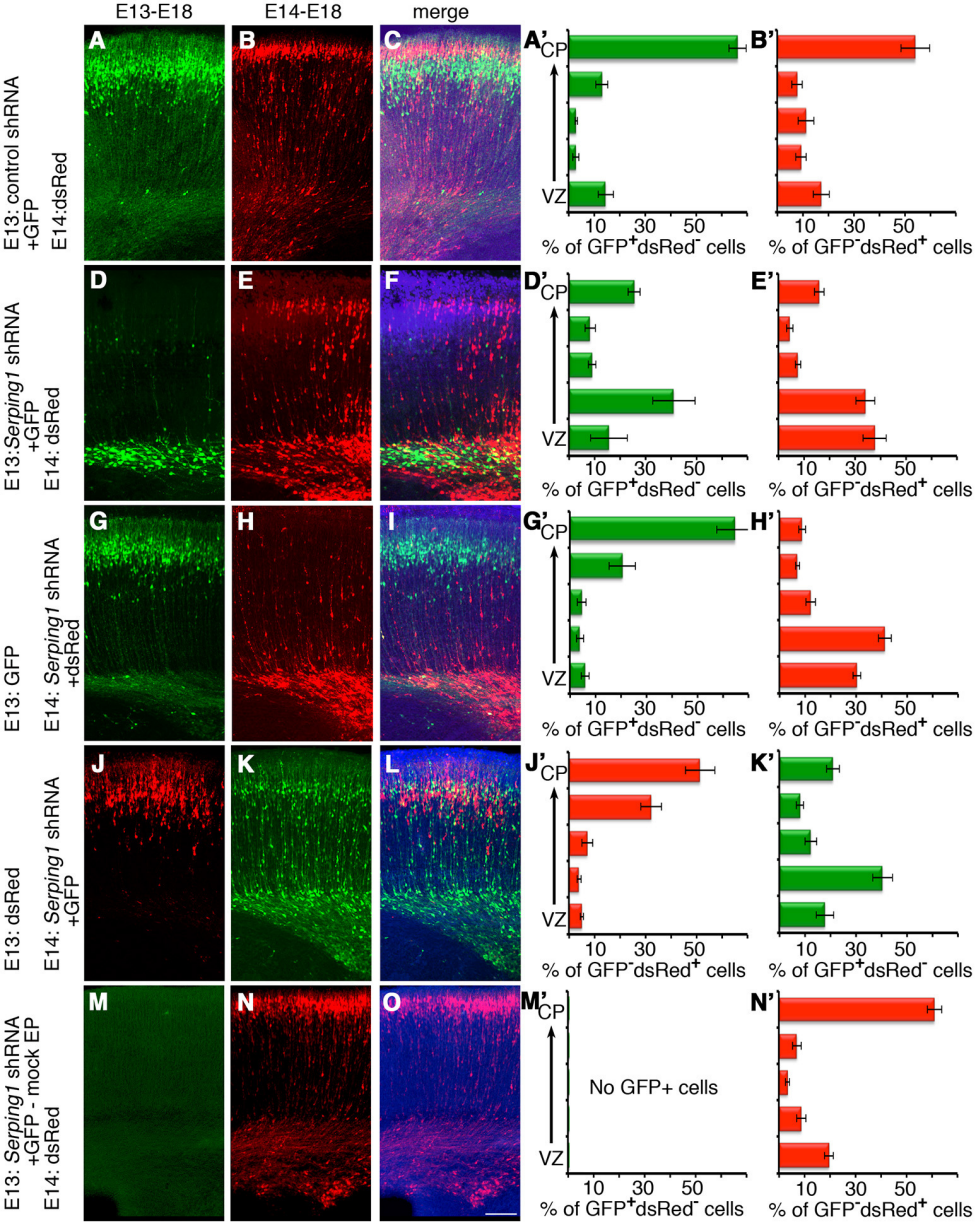
Non-cell autonomous effects of *Serping1* shRNA. **(A–F)** *Serping1* shRNA exhibits non-cell autonomous effects. Brains were *in utero* electroporated with control shRNA **(A–C)** or *Serping1* shRNA **(D–F)** together with GFP on E13, followed by electroporation with dsRed on E14. The analysis was performed on E18. GFP-positive neurons **(A,D)**, dsRed-positive neurons **(B,E)**, and merged images **(C,F)** are shown. The position of GFP-positive dsRed-negative **(A',D')** and dsRed-positive GFP-negative **(B',E')** cells was analyzed. The distribution of the dsRed-positive GFP-negative cells **(E,E')** demonstrates non-cell autonomous effect of *Serping1* shRNA (compare to **B,B'**). *n* = 6. **(G–O)** Controls of non-cell autonomous experiment. **(G–L)** The population of the neurons can be easily segregated as demonstrated by the inverse order of electroporation. Brains were electroporated with GFP on E13 and with *Serping1* shRNA with dsRed on E14 **(G–I)**, or vice versa brains were electroporated with dsRed on E13 and with *Serping1* shRNA with GFP on E14 **(J–L)**. Cells treated with shRNA demonstrated impaired migration **(H',K')**. In both conditions cells without shRNA had no defect in migration (**G,G'**,**J,J'**). **(M–O)** Control experiment demonstrates that there is no left-over of the plasmids in between electroporations. *Serping1* shRNA together with GFP were injected but not electroporated into the ventricle on E13. dsRed was electroporated on E14. On E18 there are no GFP-positive cells **(M,M')**. The migration of dsRed-positive cells was not affected **(N,N')**. The scale bar is 100 μm.

Next, the possibility that *Serping1* may affect neuronal migration in a non-cell autonomous way in addition to the cell autonomous effect observed above was examined (Figure 3). The experimental design included labeling and monitoring two distinct populations in the developing embryonic brain by consecutive electroporation. The first population was treated with shRNA (at day E13) and labeled with GFP. The position of the first population reflected cell autonomous effects. The second cell population was electroporated with a red fluorescent protein only a day later (E14) and thus its behavior is presumed to reflect non-cell autonomous effects emanating from the first (green) population. We concluded that the effect of *Serping1* knockdown was both cell autonomous and non-cell autonomous; it affected the genetically modified cells as well as the neighboring cells. Although in the control, later born cells (red) successfully migrated through the layer of previously born cells (green) treated with control shRNA (Figures 3A–C, quantification in Figures 3A',B'), the migration of the red cell population through the green cell layer, which was treated with *Serping1* shRNA, was markedly impaired (Figures 3D–F,D,E', compare Figure 3B and Figure 3B' to Figure 3E and Figure 3E', the correlation of the relative position of the red cells in B and E is -0.03). When the order was reversed, the later born *Serping1* knockdown cells did not impair the motility of earlier born control green (Figures 3G–I,G', H' the correlation of the relative position of the red cells in B and green cells in G is 0.92) or red positive cells (Figures 3J–L,J',K', the correlation of the relative position of the red cells in Figure 3B and red cells in Figure 3J is 0.76). The observed non-cell autonomous effect was not due to residual, stable *Serping1* shRNA in the cortex. *Serping1* shRNA and GFP plasmids were injected into the cortex at E13 but not electroporated, a day later, a red fluorescent plasmid was injected and electroporated. No GFP positive cells were noticed at E18 and no neuronal migration impairment was noted (Figures 3M–O,M',N'). A non-cell autonomous effect was also observed using a different approach. The cells neighboring *in utero* E13 electroporated cells were labeled at E14 by a uridine analog, and their position in the cortex was analyzed at E18. The relative distribution of the IdU labeled cells in the *in utero* electroporated side of the brain differed significantly from the relative distribution of IdU labeled cells on the non-*in utero* electroporated side of the brain (Supplementary Figure 4A–B', three out of five bins showed significant differences Supplementary Figure 4C). Taken together, our data suggest that SERPING1 participates in regulation of radial neuronal migration in a cell autonomous as well as in a non-cell autonomous fashion.

**Figure 4:**
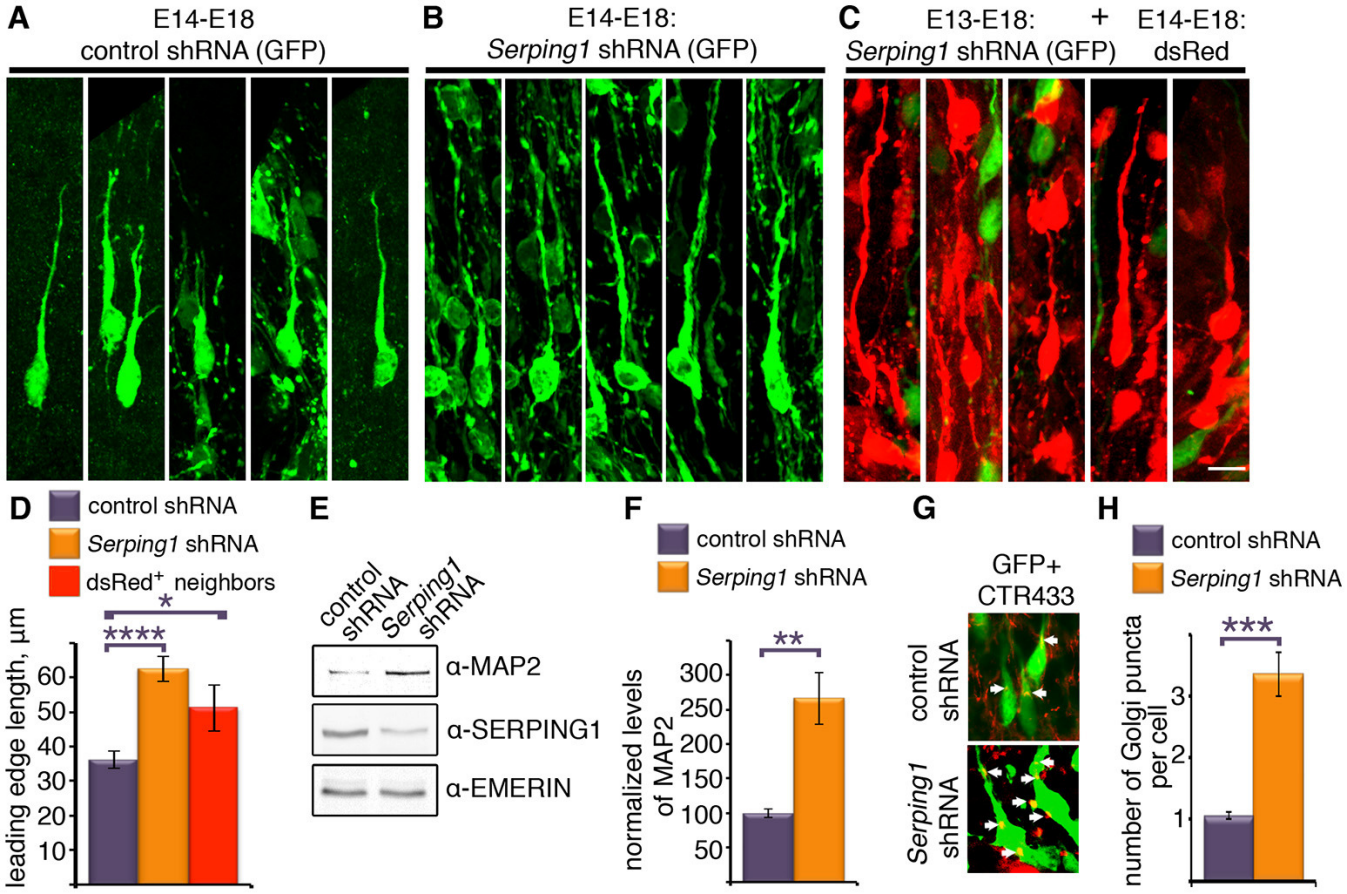
*Serping1* influences neuronal morphology in a cell-autonomous and non-cell autonomous way. **(A–D)**. Brains were *in utero* electroporated with control shRNA **(A)** or *Serping1* shRNA **(B)** together with GFP (E14-E18). Representatives of individual cells from the IZ are shown **(C)**. Brains were *in utero* electroporated with *Serping1* shRNA on E13 and with dsRed on E14. DsRed positive cells arrested in IZ (E18) are shown. The length of the leading edge was measured on high-magnification 3D reconstruction images with ImageJ software. The lengths of the leading edges were compared between the conditions **(D)**. One-way ANOVA, Turkey HSD, *n* = 16, ^*^p < 0.05, ^****^p < 0.0001. The scale bar is 10 μm. **(E,F)** Brains were *in utero* electroporated with control shRNA or *Serping1* shRNA together with GFP (E14-E17). The electroporated areas were dissected under fluorescent binocular. The dissected areas were lysed and western blotted using anti-MAP2, anti-SERPING1 and anti-EMERIN antibodies. The levels of MAP2 were normalized to EMERIN. Levels of MAP2 relative to control (in %) are presented **(F)**. *n* = 3, *Student t*-test, ^**^p < 0.01 **(G–H)**. Golgi analysis in *Serping1* knockdown compared to control. Control shRNA or *Serping1* shRNA electroporated brain sections (E14-E18) were immunostained with Golgi marker antibodies (CTR433). Immunostaining of the Golgi are presented together with GFP. White arrows show position of Golgi. The scale bar is 10 μm. Quantification of the number of Golgi clusters per cell (*n* = 20, *Student t*-test) are shown **(H)** ^***^p < 0.001.

Closer inspection of the stalled cells revealed that they exhibit long processes (Figures 4A,B). In addition, it appeared that wild-type cells that were in close vicinity to the *Serping1* shRNA treated cells also exhibited very long processes (Figure 4C). The length of the leading edge of individual cells was quantified and the leading-edge length of either *Serping1* shRNA treated cells or of their red neighbors significantly differed from the values of the controls (Figure 4D). Therefore, western blot analyses were conducted using several antibodies. Most strikingly, the levels of MAP2 increased more than 2.5-fold in *Serping1* shRNA brain lysates (Figures 4E,F). No differences were noted for phospho-DCX and phospho-ERK antibodies. MAP2 (microtubule-associated protein 2) is a prominent microtubule associated protein expressed in the developing brain (Dinsmore and Solomon, 1991). The increased levels of MAP2 are consistent with the observed morphological changes. Although the elongated *Serping1* shRNA treated cells appeared to be bipolar, immunostaining with a Golgi marker that appears as a packed cluster at the basal side of nucleus in the migrating bipolar control cells, revealed that the cells have not completed polarization. On average more than three Golgi clusters were noted in the treated cells instead of one observed in control cells (Figures 4G,H).

### *Serping1* is part of the complement pathway

**Figure 5:**
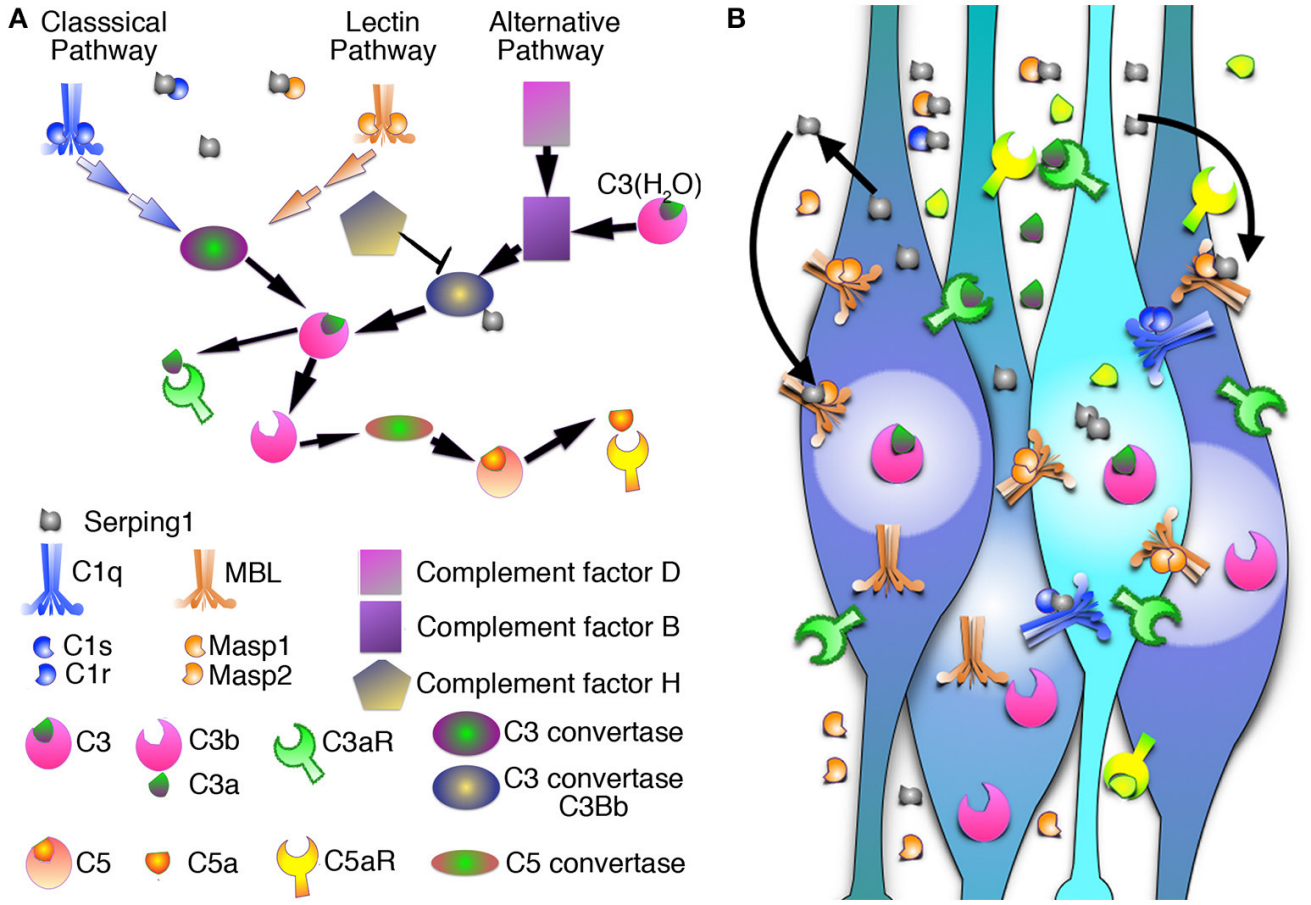
Schematic representation of the complement pathway. **(A)** Three parallel arms of the complement pathway: the classical pathway, the lectin pathway, and the alternative pathway converge on the level of the C3 that is cleaved by C3 convertase (C4bC2a in the classical and lectin pathway, or C3-H2OBb in the alternative pathway) into C3a anaphylotoxin and C3b. C3aR binds C3a. C3b is recruited to form C5 convertase, which processes inactive C5 into C5a anaphylotoxin and C5b. C5aR binds C5a. All three pathways are regulated by Serping1 or C1 inhibitor. **(B)** Schematic representation of Serping1 cell-autonomous and non-cell autonomous roles in migrating neurons. Serping1 is expressed and secreted by neurons and inhibits complement pathway by direct interaction with C1s, C1r, Masp1, and Masp2. Serping1 functions in the extracellular matrix and influences both the neuron it was secreted from and neighboring neurons.

**Figure 6:**
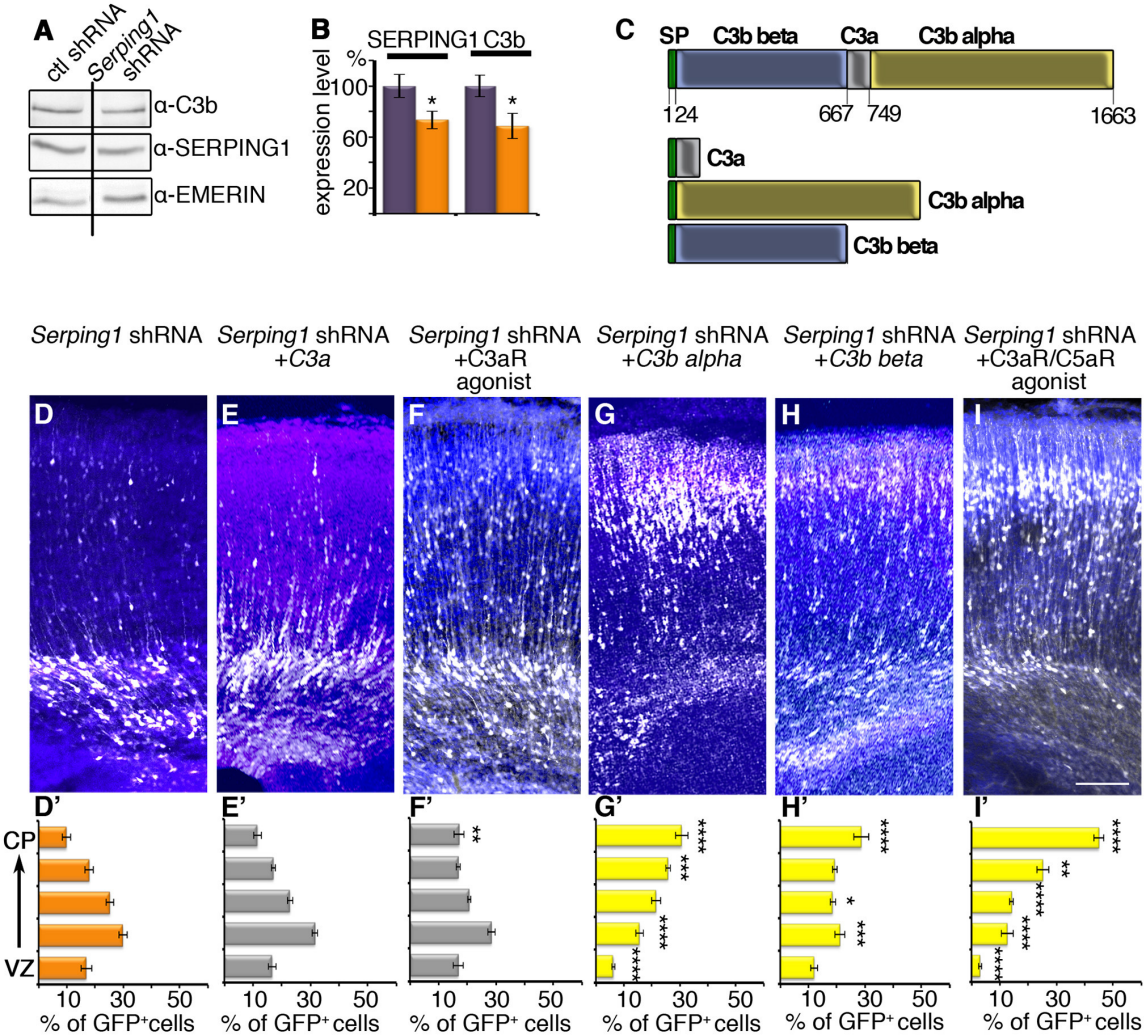
*Serping1* in the complement pathway. **(A,B)** Levels of activated C3 (C3b) are slightly reduced in the *Serping1* shRNA compared to control. Areas from the brains electroporated *in utero* on E14 with control or *Serping1* shRNA were dissected on E17 and subjected to western blot analysis with anti-C3b antibodies, anti-SERPING1 and anti-EMERIN antibodies. The levels of SERPING1 and C3b were normalized to EMERIN and quantified (*n* = 9). ^*^p < 0.05. **(C)** Schematic representation of C3. The proteolytic cleavage of C3 on aa 667 and aa 749 results in C3a peptide and C3b, composed of two subunits: C3b beta and C3b alpha. Three fragments mimicking the cleavage products of C3, all with an N-terminal signal peptide (SP, green). **(D–I)** Rescue of *Serping1* shRNA migration phenotype with C3 fragments and peptides, agonists of Complement receptors. Brains were electroporated (E14-E18) with *Serping1* shRNA alone **(D)** or together with either *C3a* **(E)**, C3aR agonist **(F)**, *C3b alpha* **(G)**, *C3b beta* **(H)**, or dual C3aR/C5aR agonist **(I)**. The scale bar is 100 μm. The distribution of neurons along the cortex in 5 bins is shown **(D'–I')**. All conditions were compared to *Serping1* shRNA. Two-way ANOVA, *n* = 11, ^*^p < 0.05, ^**^p < 0.01, ^***^p < 0.001, ^****^p < 0.0001.

Finally, we evaluated whether the developmental effects of *Serping1*, were due to its role within the complement pathway. *Serping1*, or C1 inhibitor, participates in the three activation arms of the complement system. It covalently binds and inhibits the activity of the C1r and C1s serine proteases that are involved in initiation of the classical pathway (hence the origin of the name C1 inhibitor). However, it also covalently binds and inhibits the activity of the closely related MASP1 and MASP2 proteases, which initiate the lectin pathway. It can also physically bind and functionally affect the interaction between complement factor C3b and complement factor B, and thus to interfere also with the alternative pathway(see schematic presentation in Figure 5). Therefore, *Serping1* or C1 inhibitor inhibits all the activation arms of complement. Complement activation will lead to an increase in the levels of cleaved C3, which can be detected by anti-C3b antibodies. Brain lysates from *Serping1* shRNA treated and control treated were therefore analyzed by western blot using anti-C3b antibodies (Figure 6A). Contrary to our expectations, a small yet significant reduction in the levels of C3b were noted (Figure 6B). It was postulated that pathway activation may result in rescue of neuronal migration impairment and we next queried the effect of C3 mimicry cleavage products (scheme in Figure 6C) and either the single C3a receptor (C3aR), or the dual C3aR/C5a receptor (C5aR) agonists in combination with *Serping1* shRNA. As previously observed, *Serping1* shRNA impairs neuronal migration (Figures 6D,D'). When the C3a mimicry product was expressed *in utero* together with *Serping1* shRNA no effect was observed (Figures 6E-E'). The effect of the addition of the downstream C3aR agonist corroborated this finding, which minimally affected the proper position of migrating neurons (Figures 6F,F'). Addition of the C3 mimicry cleavage products C3b alpha or less effectively C3b beta, significantly improved neuronal migration (Figures 6G–H'), suggesting that activation of C3 cleavage is required for proper neuronal migration. Furthermore, the addition of the dual C3aR/C5aR agonist completely restored neuronal positioning to control levels (Figures 6I–I'), supporting that downstream complement activation at the level of C5a is required for proper migration of pyramidal neurons to the cortical plate.

## Discussion

### The role of *Serping1* in the developing cortex

Collectively our findings suggest that *Serping1* (C1 inhibitor) is important for cortical development at several stages. At early stages of cortical development *Serping1* is important for the proliferation of neuronal stem cells. A decrease was noted in the number of cells in S phase that include both radial glia and intermediate progenitors. The effect may be transient since the total number of TBR2 positive basal progenitors, cells was not affected. *Serping1* has been reported to be expressed in the SVZ basal progenitors (Kawaguchi et al., 2008), yet our study is the first demonstrating a direct role for this gene product in neuronal stem cell proliferation. Our previous studies showed that knockdown or knockout of other components of the complement pathway affects neuronal stem cell proliferation in different ways. Knockout of C3 increased the number of mitotic intermediate progenitors (phospho-Histone positive), whereas knockdown of *Masp1* did not affect the number of progenitors in S phase (IdU positive) and knockdown of *Masp2* resulted in a statistically significant increase in the number labeled progenitors (IdU positive) (Gorelik et al., 2017). These highly variable outcomes may suggest that within neuronal stem cell progenitors there is not a linear relationship between changes in the components of the complement pathway and neuronal stem cell proliferation. Alternatively, it is possible that the spatiotemporal expression pattern in different subsets of neuronal progenitors dictates the outcome of pathway modulation. Following exit from the cell cycle, cortical neurons migrate along the processes of the radial glia to reach the cortical plate. Knockdown or knockout of *Serping1* impairs radial migration. The delay in reaching the cortical plate did not affect cell identity, and the stalled cells express CUX1 similar to the control cells. Migration delay is transient and in the postnatal brain, the cells eventually reach the cortical plate. Interestingly, *Serping1* knockdown affected not only the manipulated cells but also their neighbors; thus it is working both in a cell autonomous and a non-cell autonomous way. The cell autonomous and non-cell autonomous effect was noticed not only on the position of the cells in the developing cerebral cortex, but was also seen in the cellular morphology of the neighboring cells. *Serping1* knockdown cells exhibited long leading processes, which were also observed in near neighbors. These extended processes may be due to elevated levels of the microtubule-associated protein MAP2, which was detected by western blot analysis of brain lysates. The non-cell autonomous effects may be of particular interest when considering the possibilities of somatic mutations in the developing brain. The notion that somatic mutations play an important role in human disease has been widely accepted in case of cancer, in which even genetic mutations usually require a second somatic hit to initiate the development of tumors (Hanahan and Weinberg, 2000). Furthermore, conceptual progress in the last decade allows us to assimilate that participation in disease progression is not restricted to the very cells that are mutated, but also to the adjacent “healthy” tissue which changes its properties as a consequence of the presence of the mutated cells (review Hanahan and Weinberg, 2011). Emerging evidence suggest that somatic mutations may also participate in brain diseases (review Gleeson et al., 2000). Perhaps one of best-known examples is *DCX*, an X-linked gene (des Portes et al., 1998; Gleeson et al., 1998), in which mutations result in lissencephaly in males and a “doublecortex” phenotype in females due to random X-inactivation. On the other hand, somatic mutations in the same gene in males result in the “doublecortex” phenotype (Gleeson et al., 2000). *Serping1* non-cell autonomous effects then adds on to another gene characterized from the same screen *Atx*, which also exhibited non-cell autonomous effects (Greenman et al., 2015).

### The role of Serping1 in migrating neurons in relation to the complement pathway

Our unbiased screen for molecules, which participate in non-cell autonomous regulation of neuronal migration revealed Serping1 as a molecule, which is differentially expressed and allowed us to uncover an unexpected role of the innate immune complement pathway in the regulation of neuronal migration (Gorelik et al., 2017). There may be several possible reasons as for why this pathway has been recruited to function in a non-cell autonomous fashion in the developing brain. The pathway consists of a cascade of proteases, coupled with many inherent regulatory steps. Many of the pathway components are secreted or bound to the cell surface, ideal for cell-cell communication (reviews Walport, 2001; Fujita, 2002; Rutkowski et al., 2010; Stephan et al., 2012). As opposed to cell destruction induced by the complement pathway in response to pathogens (Fujita, 2002), this pathway has a vital role in migrating neurons. Based on our data, we suggest that activation of the pathway may result in reshaping of cells, possibly via partial opsonization, or tagging of multipolar neurites, enabling the polarity transition of neurons toward successful migration along radial glia. Our experimental results also indicate that the developing brain uses components of the complement in a non-orthodox way, as knockdown of Serping1 did not trigger activation of the pathway. However, the C3a peptide, or a C3a receptor agonist did not rescue neuronal positioning following Serping1 shRNA mediated knockdown. These results are different from those obtained following knockdown of *Masp2, C3*, or in *C3* KO mice, where either the single or the dual agonists rescued migration (Gorelik et al., 2017). C3a has been demonstrated to act as a chemo-attractant during collective cell migration of neural crest cells (Carmona-Fontaine et al., 2011) and enteric neural crest cells (Broders-Bondon et al., 2016). In case of in *Serping1* shRNA treatments, fragments mimicking C3 cleavage (C3b beta and alpha) rescued the migration impairment neurons. Moreover, our data indicate that signaling is likely to be also transmitted through the complement C5a receptors, which when activated, completely rescued neuronal migration deficits observed in the case of *Serping1* knockdown. It should be noted that this study could not distinguish between a role for C5a receptor 1 or C5a receptor 2 (C5L2). However, it has been previously demonstrated that perturbations in C5a receptor 1 signaling during rodent brain development can result in select neuronal defects (Benard et al., 2008; Denny et al., 2013; Coulthard et al., 2017), possibly indicating a predominant role for this receptor subtype in embryonic development (Hawksworth et al., 2014). Regardless, our study adds to the evidence for widespread roles for complement fragments C3a and C5a in development (Hawksworth et al., 2016).

The complement system is indeed a complex pathway with three “linear” arms that converge but also interconnect at several point. We therefore cannot be confident about the exact regulatory mechanism and possible positive and negative feedback loops in which *Serping1* relates to the pathway in the context of progenitor proliferation and neuronal migration. In many respects, this process exhibits analogies to the previously described role of the complement pathway in successful elimination of excess numbers of synapses, which is developmentally regulated (Stevens et al., 2007; Schafer et al., 2012). Other components of the immune system, such as the major histocompatibility complex (MHC) and many others, have been shown to play a role in the developing brain (reviews Boulanger and Shatz, 2004; Boulanger, 2009). Furthermore, the uniqueness and unexpected results following Serping1 knockdown may be due to additional activities of this potent molecule beside the complement system (Davis et al., 2008), which should be investigated in the future.

## Methods and materials

### Plasmids and primers

The following shRNA constructs were cloned into pSuper vector (Brummelkamp et al., 2002): *Dcx* shRNA1 (5′- GCTCAAGTGACCACCAAGGCTAT) (Bai et al., 2003); *Dclk* shRNA1 (5′- GGTTCGATTCTAC AGAAAT) (Koizumi et al., 2006). The following shRNA was purchased from OpenBiosystems (ThermoScientific) in pLKO.1 vector: *Serping1* shRNA (5′- CCTGACGATGCCTCATATAA). The control shRNA plasmid used is pLKO.1-TRC control (Addgene) containing non-hairpin 18 bp sequence (5′- CCGCAGGTATGCAACGCG).

A plasmid containing the complete coding sequences of *Serping1* (BC002026.1) was purchased from OpenBiosystems (ThermoScientific) and subcloned into pCAGGS plasmid. This plasmid was used as a basis for creating shRNA resistant plasmids. 4 mismatches with the shRNA sequence were inserted in the original sequences by PCR using the following primer: 5′- AAGCTCGAGCTGTCCAAATTCCTGCCCACTTACCTACCA TGCCACACATAAAGT (*Serping1*).

A plasmid containing the complete coding sequences of *C3* (BC043338.1) was purchased from OpenBiosystems (ThermoScientific). C3a was subcloned from the C3 plasmid with following primers: 5′- ATATGGCTAGCTCAGTACAGTTGATGGAAA and 5′- ATAGCGGCCGCTCACCTGGCCAGGCCCAGCACG. C3b beta was subcloned from the C3 plasmid with following primers: 5′- ATATGGCTAGCATCCCCATGTATTCCATCATT and 5′- ATAGCGGCCGCTCAGGCTGCTGGCTTGGTGCACTC. *C3b alpha* was subcloned from the *C3* plasmid with following primers: 5′- ATATGGCTAGCATCCCCATGTATTCCATCATT and 5′- ATAGCGG CCGCTCAGTTGGGACAACCATAAAC. The C3 fragments were subcloned into the pCAGGS plasmid that contained C3 signal peptide (5′- ATGGGGGACCAGCTTCAGGGTCCCAGCTACTAGTGCTACTGCTGCTGTTGGCCAGCTCCCCATTAGCTCTGGGG).

For Real-Time PCR of *Serping1* the following primers were used: 5′- GCCCAATTCGATGACCATAC and 5′- AAGTTGGTGCTTTGGGAACA; 5′- GCCCAATTCGATGACCATAC and 5'-AGTGGGGTTGAGAGCCTTTT; 5′ –TTCCCTGAAAGAGATGACTCCTGGA and 5′- CGTTGGCTACTTTACCCATGGTGTC; 5′- TGGAGTCCCCCAGAGCCTACA and 5′- GAGGAGGCTGGCAATGCTGA. *29rps* primers were used as a reference: 5′- GTATTTGCGGATCAGACCGT and 5′- CTGAAGGCAAGATGGGTCA.

For PCR of *Serping1* cDNA the following primers were used: 5′- AGAGAGCTTCCCTGAAAGAGATG and 5'- TGAGGAGGCTGGCAATGCTGA.

### Antibodies

Rabbit anti SERPING1/C1INH (Santa Cruz, H-300, 1:250), rabbit anti EMERIN (Santa Cruz, FL-254, 1:1,000), mouse anti-MAP2 (Sigma-Aldrich, HM-2, 1:500), rat anti C3b antibodies (Hycult Biotech, 1:500, HM1065) were used for western blotting.

The following antibodies were used for immunostainings: chicken anti TBR2 (Millipore, 1:100, AB15894), rabbit anti CUX1 (anti CDP, Santa Cruz, 1:100, SC-13024), chicken anti TBR1 (Millipore, 1:100, AB2261), mouse anti IdU-B44 (BD Biosciences, 1:200, 347580), chicken anti GFP (Abcam, 1:1000). Mouse CTR433 antibodies (1:50, Jasmin et al., 1989), a Golgi marker, was kindly provided by Dr. Michel Bornens (Institute Curie, Paris, France).

### Complement agonist peptides

The selective C3aR agonist, WWGKKYRASKLGLAR (“super-agonist” Wu et al., 2013), and a C5aR agonist, YSFKPMPLaR (“EP54” Woodruff et al., 2001) were synthesized as previously described (Woodruff et al., 2001; Wu et al., 2013). It should be noted that the C5aR agonist also activates C3aR (Scully et al., 2010) and thus is described herein as a dual C3aR/C5aR agonist. The agonists (1 μg/mg) were injected to the ventricles of the embryos together with the indicated plasmids.

### Animals

Animal protocols were approved by the Weizmann Institute IACUC and were carried out in accordance with their approved guidelines. ICR mice were purchased from Harlan laboratories. Male and female embryos were used in the study.

### CRISPR/Cas9 knockout generation

Cas9 plasmid and plasmids encoding guide RNAs were purchased from the University of Utah Mutation Generation lab. The following oligonucleotides were used for construction of gRNA vectors:

1. Serping1: 5′ ACACCGGCTACACTGGTTGTTGGCCG and 5′ AAAACGGCCAACAACCAGTGTAGCCG (location 2:24847967-24847990: + strand);

*In vitro* transcribed Cas9 RNA(100 ng/ul), and sg RNA(50 ng/ul), were injected into one cell fertilized embryos isolated from superovulated CB6F1 hybrid mice mated with CB6F1 males Harlan Biotech Israel Ltd. (Rehovot, Israel). Injected embryos were transferred into the oviducts of pseudopregnant ICR females as previously described (Wang et al., 2013). For migration analysis the pregnant mice were subjected to IdU injection at E14.5 and sacrificed at E18. Genomic DNA from the treated embryos was analyzed for mutations in the mutated genes using High Resolution Melt (HRM) analysis and confirmed by Sanger sequencing. For the proliferation analysis the mice from established mouse line were used. The genotype verification was performed with the following primers: 5′- TTCCCTGAAAGAGATGACTCCTGGA and 5′- CGTTGGCTACTTTACCCATGGTGTC.

### IdU injection

The thymidine analog iododeoxyuridine (IdU) was injected intraperitoneally (0.01 ml of 5 mg/ml IdU solution per gram body weight) into pregnant mice at the indicated time points.

### EdU labeling and click chemistry

For labeling cells in S phase, pregnant mice (E14) were injected with (50 mg/gr body weight) 5-ethynyl-2′-deoxyuridine (EdU) solution and were scarified 30 min post-injection. The brains were removed and fixed in 2.5% PFA-PBS overnight, washed and cryoporotected by immersion in 30% sucrose-PBS solution. The cryosections (10 μm) were pretreated in boiling sodium citrate buffer (10 mM, pH 6) for 30 min. The click reaction was performed with Cy3 azide (2.5 μM) in the PBS-based buffer containing 100 mM Tris-HCl, 1 mM CuSO_4_, and 100 mM ascorbic acid. This reaction was followed by a click reaction with a non-fluorescent molecule (Phenylthiomethyl-Azide 20 mM, SIGMA). After treatment with 10 mM ascorbic acid and 4 mM CuSO_4_, followed by incubation with 20 mM EDTA, the relevant immunostainings were performed.

### *In utero* electroporation

Plasmids were transfected by *in utero* electroporation using previously described methods (Saito and Nakatsuji, 2001; Tabata and Nakajima, 2001). Prior to surgery the animals were injected with buprenorphine (2 mg/Kg BW, subcutaneously). Pregnant ICR mice at E14.5 days post-gestation (E14), were anesthetized by injection of ketamine 10%/xylazine 20 mg/ml (1/10 mixture, 0.01 μl per g of body weight, intraperitoneally), alternatively Isofluran anesthesia was used. The uterine horns were exposed, and plasmid mixed with Fast Green (2 μg/μl, Sigma) were microinjected through the uterus into the lateral ventricles of embryos by pulled glass capillaries (Sutter Instrument, Novato, CA). The concentration of plasmids was 0.5 μg/μl for pCAGGS-GFP, 2 μg/μl for shRNA construct and 1–1.5 μg for overexpression plasmids. Electroporation was accomplished by discharging five 41 mV 50 ms long pulses with 950 ms intervals, generated by a NepaGene electroporator. The pulses were delivered using 10 mm diameter platinum plated tweezers electrodes (Protech international Inc., San Antonio, TX) placed at either side or the head of each through the uterus. Animals were sacrificed 4 days after electroporation at E18.5 (E18). Embryos with well-distinctive positive GFP signal in cortex visible through fluorescent binocular were intracardially perfused using 4% paraformaldehyde–phosphate buffered saline (PFA-PBS). Embryos with dotted, double hit or hit outside the cortex were not included in the study. Brains were post-fixed overnight and sectioned (60 μm; vibrotome, Leica). For examination of long-term effects of the treatments *in utero* electroporation was performed at E14, the mice delivered and the pups of postnatal day 8 (P8) were used for the experiments. For proliferation experiments embryos were *in utero* electroporated on E13.5 (E13) with 7 mm electrodes (39 mV pulses). IdU was injected in 24 h after electroporation for 30 min. Post-fixed brains were cryopreserved in sucrose and cryosections (10 μm) were used for proliferation analysis. For double electroporation the first *in utero* electroporation was performed at E13.5 with 7 mm electrodes (39 mV pulses). The second electroporation was performed 24 h later as described above.

### Immunocytochemistry

Floating vibratome sections (60 μm) were permeabilized using 0.1% Triton X-100 and blocked in blocking solution (PBS, 0.1% Triton X-100, 10% HS; 10% FBS) for 60 min. Antibodies were incubated in blocking solution over night at 4°C. After washing, appropriate secondary antibodies (Jackson ImmunoResearch) were diluted in blocking solution, and incubated for 2 h at room temperature. Slices were mounted onto glass slides using Aqua Polymount (Polysciences). For IdU immunostainings (E18) the brain slices were pretreated with HCl (30') followed by neutralization with borate buffer. For IdU immunostainings (E13 and E14) the cryosections (10 μm) were used. Antigen retrieval procedure was performed by boiling slides in sodium citrate buffer (10 mM, pH 6) for 30 min.

### Microscopy, quantification, and statistical analyses

Images were taken using confocal microscopy (LSM780, LSM800 Zeiss), equipped with Axio Observer Z1 microscope, and imaged with either Plan-apochromat 20x/0.8, or Plan-apochromat 40x/1.2, or Plan-apochromat 63x/1.4 oil objectives. The scaling data are 0.624X0.624 μm per pixel for 20X magnification, 0.312X0.312 μm per pixel for 40X magnification, and 0.198X0.198X0.51 μm per voxol for 60X magnification. The images were processed by ZEN software and/or Imaris software.

Cell count, positioning and colocalization analyses were performed using Imaris software (Bitplane Inc., Zurich, Switzerland, Imaris core module). At least three brains were analyzed for each treatment. Four representative slices from each brain were chosen for analysis. The size of the area of interest was determined and preserved per each experiment. For each slice the area of interest was positioned so that the center of the electroporated area is in the center of the area of interest. For the cell count and positioning the relevant channel of an area of interest was analyzed with “Spots” module of Imaris, every spot labeling approximate center of the cell body. The “y” position of all the dots was analyzed by Microsoft Excel Histogram tool. The data were presented in percentages out of total analyzed cells per bin. For Figure 1B an average of 135.6 ± 8.3 (control shRNA) and 129 ± 15.4 (Serping1 shRNA) GFP-positive cells were analyzed per each slice. For Figure 2D an average of 490.8 ± 44.1 (control shRNA), 704 ± 51.8 (Serping1 shRNA), and 662.1 ± 52.8 (Serping1 shRNA +Serping1res) GFP-positive cells were analyzed per slice. For Figure 2G an average of 249.5 ± 12.4 (WT) and 259.5 ± 13.6 (Serping1 KO) IdU-positive cells were analyzed per slice. For Figure 3 an average of 175.6 ± 14.3 (Figure 3A'), 180 ± 12.5 (Figure 3B'), 267.6 ± 37 (Figure 3D'), 182 ± 17.2 (Figure 3E'), 174.6 ± 19.5 (Figure 3G'), 137.1 ± 11.5 (Figure 3H'), 111.7 ± 9.3 (Figure 3J'), 166 ± 13.3 (Figure 3K'), 112 ± 21 (Figure 3N') GFP- or dsRed-positive cells were analyzed per slice. For Supplementary Figure 4C an average of 275.1 ± 24.5 (electroporated side) and 226.7 ± 30.9 (non-electroporated side) cells were analyzed per slice. Statistical analysis was performed by Student's or Welch's *t*-tests or one-way or two-way analysis of variance (ANOVA) followed by Bonferroni multiple comparison analysis, using PRISM 7 for Mac (GraphPad software). Error bars represent standard error. For the measurement of leading edge length high resolution z-stack images were collected of the relevant slices. The analysis was performed with the ImageJ program.

### Real-time qRT-PCR

For confirmation of shRNA efficiency neurospheres from E13.5 were grown in Neurobasal medium (Gibco) supplemented with B27, glutamax, gentamicine, EGF (20 ng/ml), bFGF (20 ng/ml), and heparin for 2 days. The cells were transfected by NEPA21 electroporator (Nepagene) according to manufacturer's instructions. The cells were grown for additional 48 h and collected for RNA isolation (TRI reagent, Sigma). After Dnase treatment (Sigma), first-strand cDNA synthesis was done using M-MLV RT (Promega). Relative levels of Serping1 expression were normalized to the 29rps gene. Real-time PCR with SYBR FAST ABI qPCR kit (Kapa Biosystems) was performed using StepOnePlus Real-Time PCR System (Applied Biosystems). E13, E14, E16, E18 cortices (*n* = 6 for each time point) were dissected in cold PBS and fast-freeze in liquid nitrogen. RNA preparation and Real-time RT-PCR were performed as described above.

### Western blot

Brains were *in utero* electroporated with control shRNA or Serping1 shRNA together with GFP (E14-E17). The electroporated areas were dissected under fluorescent binocular. The dissected areas were homogenized in lysis buffer (50 mM Tris-HCl pH7.5; 150 mM NaCl; 1 mM EDTA; 1 mM EGTA; 1% Triton X-100) supplemented with protease inhibitor cocktail (Sigma). Fifty microgram of total protein was mixed with SDS sample buffer, separated by SDS-PAGE and subjected to western blot analysis with the indicated antibodies.

## Author contributions

AG and TS planned, conducted experiments, analyzed data, and wrote the manuscript. TW contributed receptor agonists, planned experiments, and wrote the manuscript. OR planned experiments, analyzed the data, and wrote the manuscript.

### Conflict of interest statement

The authors declare that the research was conducted in the absence of any commercial or financial relationships that could be construed as a potential conflict of interest.
