# Supplementary material for: Serping1/C1 Inhibitor Affects Cortical Development in a Cell Autonomous and Non-cell Autonomous Manner
Source: Front Cell Neurosci. 2017 Jun 16;11:169. doi: 10.3389/fncel.2017.00169 (PMC5472692; doi:10.3389/fncel.2017.00169)
Supplement: Supplementary file 1 [file DataSheet1.DOCX]

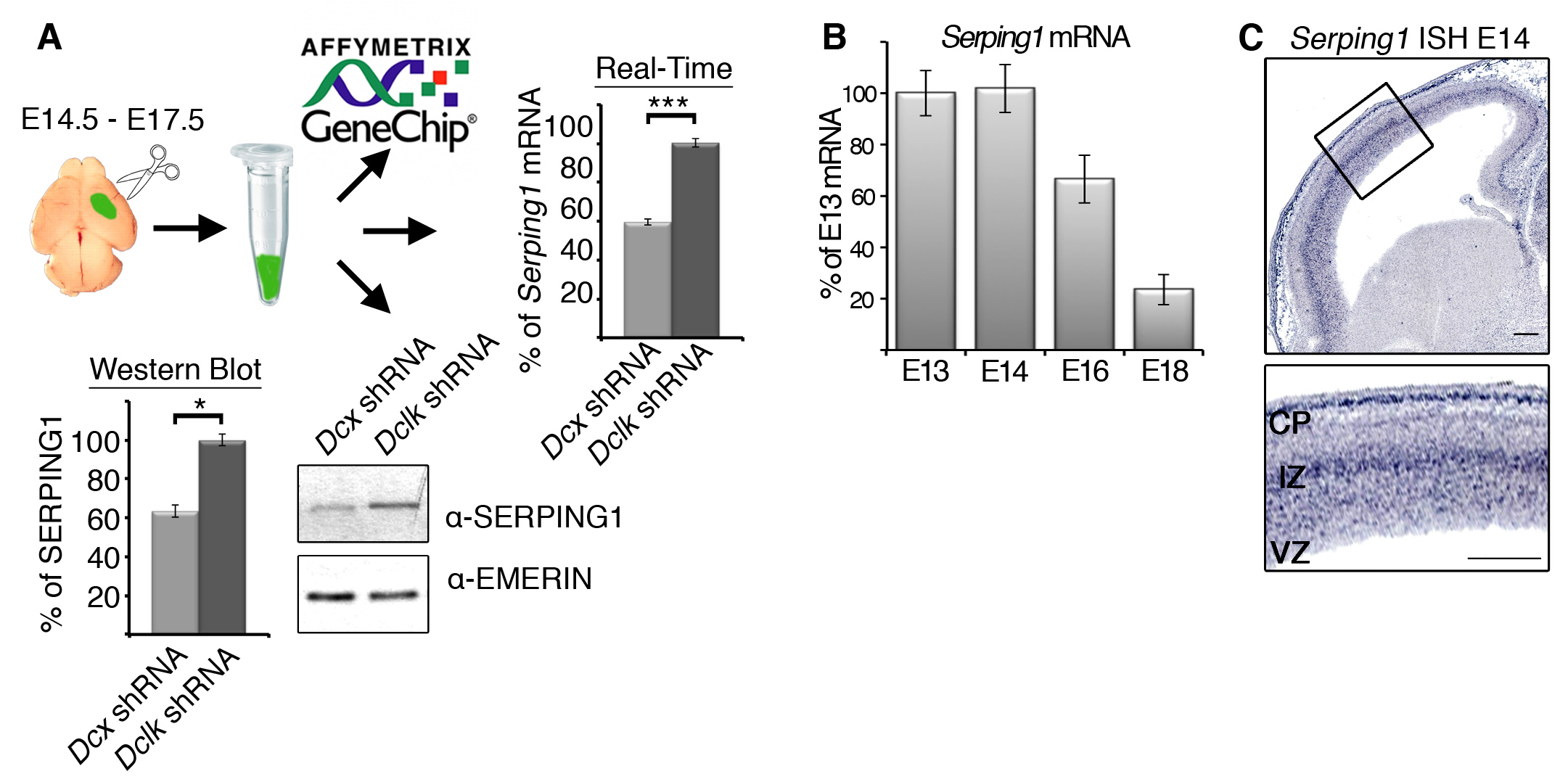


Supplementary figure 1. *Serping1* is expressed in the developing cortex and shows differential expression following knockdown of either *Dcx* or *Dclk*. (A) Embryos were electroporated *in utero* on E14 with *Dcx or Dclk* shRNA; on E17 the areas with stalled electroporated cells were dissected. RNA was prepared from 5 embryos for an Affymetrix array. The same experimental procedure was followed for preparation of RNA for Real-Time experiments and for preparation of protein lysates for western blot analysis. *Serping1* mRNA levels in the *Dcx* shRNA and *Dclk* shRNA were normalized to ribosomal protein *29rps* and are presented in the graph as % of *Serping1* mRNA level in *Dclk* shRNA treatment (n=6). The protein levels of SERPING1 in *Dcx* or *Dclk* shRNA were analyzed by western blot (n=5), normalized to levels of EMERIN and presented in the graph as % of SERPING1 levels in the *Dclk* shRNA treatment. (B) Real-time qRT-PCR showing the relative mRNA expression pattern of *Serping1*. For each time-point (E13, E14, E16, E18) cortices from 6 different embryos were used. Expression data was normalized to the expression level of the ribosomal protein *29rps*. The expression in each day is presented as % of the relative expression observed at E13 of each gene. (C) *In situ* hybridization of *Serping1* adapted from Genepaint database. E14.5, sagittal section. The scale bars are 200 μm.
