# Supplementary material for: Serping1/C1 Inhibitor Affects Cortical Development in a Cell Autonomous and Non-cell Autonomous Manner
Source: Front Cell Neurosci. 2017 Jun 16;11:169. doi: 10.3389/fncel.2017.00169 (PMC5472692; doi:10.3389/fncel.2017.00169)
Supplement: Supplementary file 2 [file DataSheet2.DOCX]

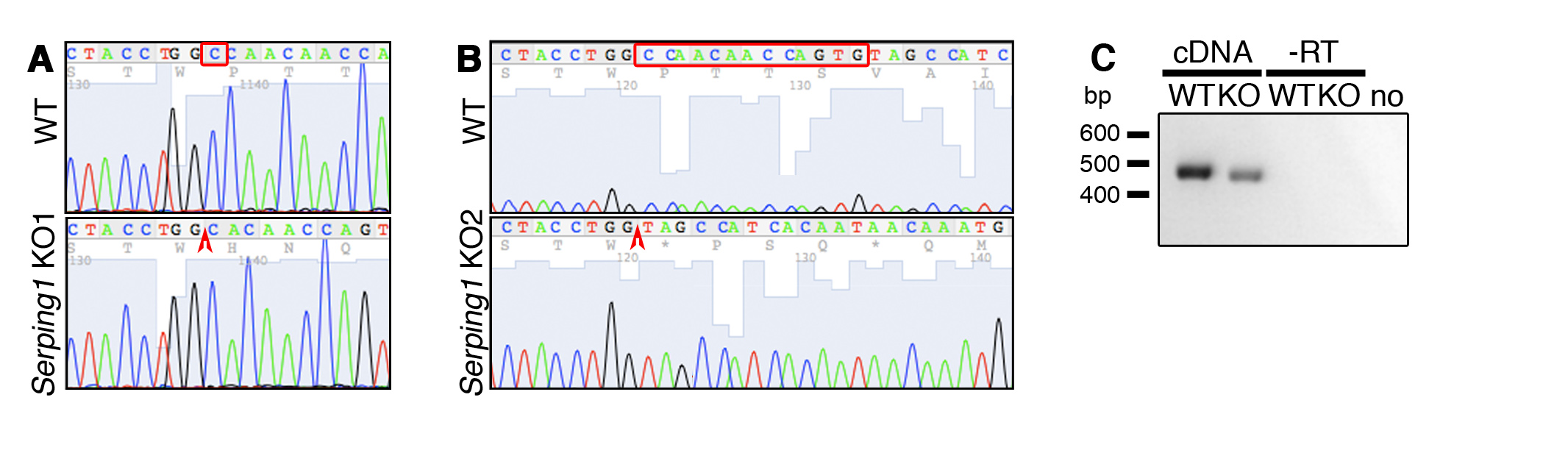


Supplementary figure 2.

(A-B) Sequences derived from two *Serping1* KO compared to WT. CRISPR-Cas9 gene edited DNA showed one (A) and thirteen (B) base-pair deletion, respectively, leading to frame-shift mutations and premature termination. The deletion in the second *Serping1* KO was verified on the RNA level (C). RNA from KO and littermate WT pups was purified and first-strand cDNA was synthesized. PCR with primers flanking the deletion shows smaller product in KO. The conditions without reverse-transcriptase and no DNA were used as control. Deletion on the RNA level was confirmed by cDNA sequencing.
