# Supplementary material for: Serping1/C1 Inhibitor Affects Cortical Development in a Cell Autonomous and Non-cell Autonomous Manner
Source: Front Cell Neurosci. 2017 Jun 16;11:169. doi: 10.3389/fncel.2017.00169 (PMC5472692; doi:10.3389/fncel.2017.00169)
Supplement: Supplementary file 3 [file DataSheet3.DOCX]

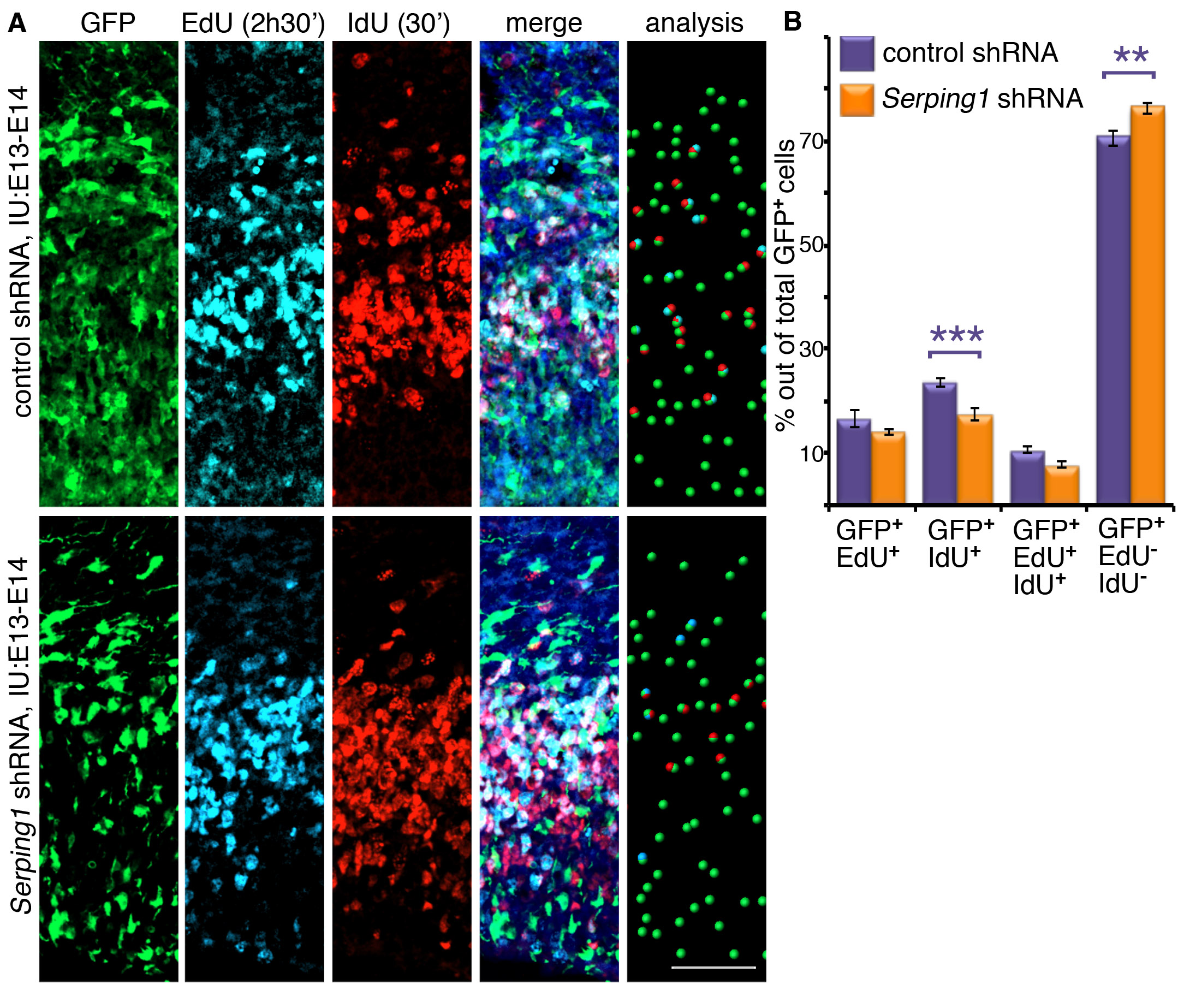


Supplementary figure 3.

(A-B) Embryonic brains were *in utero* electroporated with control shRNA or *Serping1* shRNA at E13 and at E14 were labeled with EdU (2hours 30minutes) followed by IdU (30 minutes). The brains were cryosectioned, EdU was detected by Cu(I)-catalyzed [3 + 2] cycloaddition reaction followed by immunostainings with anti-IdU antibodies. GFP labeled the electroporated cells. IMARIS software was used to count the total GFP-positive cells; GFP- and EdU-positive cells; GFP- and IdU-positive cells GFP-, EdU- and IdU-positive cells; GFP-positive EdU- and IdU-negative cells. The relative proportion of each group to the total number of GFP-positive cells was calculated (B, Two-way ANOVA, n=7, **, p<0.01, ***, p<0.001). The scale bar is 50 μm.
