# Supplementary material for: Serping1/C1 Inhibitor Affects Cortical Development in a Cell Autonomous and Non-cell Autonomous Manner
Source: Front Cell Neurosci. 2017 Jun 16;11:169. doi: 10.3389/fncel.2017.00169 (PMC5472692; doi:10.3389/fncel.2017.00169)
Supplement: Supplementary file 4 [file DataSheet4.DOCX]

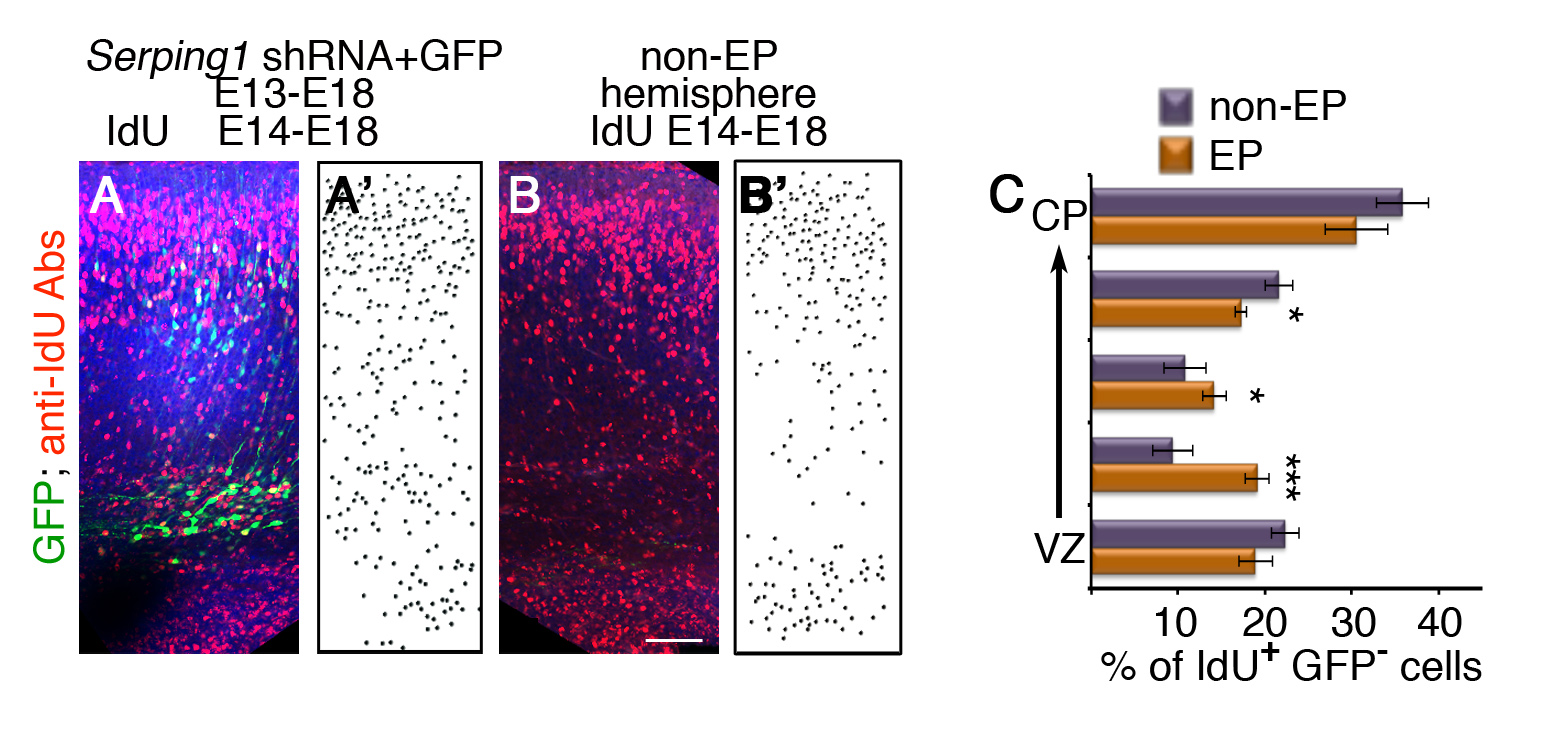


Supplementary figure 4.

Non-cell autonomous effect of *Serping1* shRNA is demonstrated by an additional approach. *Serping1* shRNA was electroporated on E13. The IdU was injected on E14. The position of IdU-labeled cells was analyzed on E18. (C) A graph compares the distribution of the IdU-positive GFP-negative cells (A,A’) with the distribution of the IdU-positive cells of non-electroporated hemisphere (B,B’). (A’,B’) Dots (IMARIS software) represent IdU-positive GFP-negative cells as used for analysis. EP, electroporated; non-EP, non-electroporated (n=7). ***,** p<0.05; ***, p<0.001. The scale bar is 100 μm.
